# Antifungal activity of selected essential oils against *Fusarium culmorum* and *F. graminearum* and their secondary metabolites in wheat seeds

**DOI:** 10.1007/s00203-019-01673-5

**Published:** 2019-05-23

**Authors:** Adam Perczak, Daniela Gwiazdowska, Katarzyna Marchwińska, Krzysztof Juś, Romuald Gwiazdowski, Agnieszka Waśkiewicz

**Affiliations:** 1 Department of Chemistry, Poznań University of Life Sciences, Wojska Polskiego Street 75, Poznań, 60-625 Poland; 2 Department of Natural Science and Quality Assurance, Faculty of Commodity Science, Poznań University of Economics and Business Poznań, Niepodległości Avenue 10, Poznań, 61-875 Poland; 3 Department of Pesticide Investigation, Institute of Plant Protection, National Research Institute, Władysława Węgorka Street 20, Poznań, 60-318 Poland

**Keywords:** Antifungal activity, Essential oils, *Fusarium* spp., Ergosterol, Zearalenone, Trichothecenes, Wheat grain

## Abstract

Essential oils (EOs) are products of plant origin and include mixtures of different chemical compounds. These volatile substances have many interesting properties, including antifungal properties. Fungi may develop under field conditions on crops such as wheat or corn and are able to synthesize mycotoxins, which adversely affect livestock and human health. In the present study, selected EOs were used to inhibit the growth of *Fusarium graminearum* and *F. culmorum* and reduce the concentrations of mycotoxins in wheat grain. The EOs significantly inhibited the growth of tested *Fusarium* species (90.99–99.99%), as determined based on ergosterol quantitative analysis. Only the addition of orange oil to *F. culmorum* exhibits a different inhibition capacity (68.13%). EO application resulted in a large reduction in zearalenone content (99.08–99.99%); only in the case of orange oil application was the reduction estimated at approximately 68.33%. However, all EOs provided a significant reduction in the concentration levels of group B trichothecenes (94.51–100%). It can be concluded that EOs inhibit the growth of fungi of the genus *Fusarium* and reduce concentration levels of the mycotoxins zearalenone and group B trichothecenes.

## Introduction

Several species of the *Fusarium* genus, including *Fusarium culmorum* and *F. graminearum*, are causative agents of severe plant diseases that are responsible for significant economic losses in crops worldwide every year (Scherm et al. 2013; Ellis and Munkvold 2014; Avanço et al. 2017). Moreover, their secondary metabolites, mycotoxins, are characterized by a wide spectrum of toxic effects (carcinogenic, mutagenic, teratogenic or oestrogenic effects), causing acute and chronic diseases in animals and humans (Zain 2011; da Cruz et al. 2013; Assunção et al. 2016). Mycotoxins are low molecular mass compounds; however, their chemical structures vary considerably. The dominant mycotoxins produced by *F. culmorum* and *F. graminearum* are zearalenone (ZEA) and deoxynivalenol (DON) and their derivatives; these mycotoxins are often identified in different cereals contributing to reduction in grain quality (Waśkiewicz et al. 2008; Goliński et al. 2010; Döll and Dänicke 2011; Zaied et al. 2012; Covarelli et al. 2015; Franco et al. 2018; Piacentini et al. 2018).

Due to their harmful effects on animal and human health as well as the need to ensure food and feed safety, there is a constant need to control fungal growth and mycotoxin production in crops. Although integrated plant protection includes a combination of various strategies, chemical fungicides still play the most significant role in the growth control of mycotoxigenic fungi. However, studies on pesticide toxicity based on the databases of the EPA, IARC, WHO, and Pesticide Action Network indicate that synthetic fungicides may cause some adverse (carcinogenic, endocrine disrupting, reproductively and developmentally toxic, acutely toxic) effects on humans (Mesnage et al. 2014; Nicolopoulou-Stamati et al. 2016). Moreover, these compounds have negative influences on the environment, including water, soil and air contamination, as well as toxic effects on non-target organisms (da Cruz et al. 2013). Therefore, in recent years, there has been increasing interest in searching for biological antifungal agents to replace synthetic pesticides. Among natural antimicrobial products, particularly interesting are plant products such as essential oils (EOs), which are some of the most promising phytochemicals and can be used for the preservation of cereals and their products (Singh et al. 2010; Boukaew et al. 2017; Foltinová et al. 2017; Gakuubi et al. 2017).

EOs are aromatic, complex, volatile, oily liquids obtained from different parts of plants (leaves, bark, stems, seeds, roots, flowers, buds, and fruits) belonging to families such as *Alliaceae*, *Lamiaceae*, *Pinaceae*, *Apiaceae*, *Rutaceae*, and *Lauraceae* (Bozin et al. 2006; Tumen et al. 2010; Shannon et al. 2011; Solorzano-Santos and Miranda-Novales 2012; Calo et al. 2015). EOs are mixtures of over 20 groups of chemical compounds, such as terpenes, alcohols, acids, esters, epoxides, aldehydes, ketones, amines, and sulfides. Their composition depends on several factors, including species and part of the plant, geographic region, agriculture method, and extraction method (Feng and Zheng 2007; Bakkali et al. 2008; da Cruz et al. 2013; Raut and Karuppayil 2014).

A number of scientific investigations have proved the antimicrobial activity of EOs derived from various herbs and plants, including their biological activity towards many fungal plant, animal and human pathogens (Lang and Buchbauer 2012; Boire et al. 2016; Grata 2016; Kumar et al. 2016; Gabriel et al. 2018). The antifungal mechanism of action of EOs is not yet completely understood. It is worth mentioning that terpenoids and phenolics, which are major components of EOs, play a crucial role in EO antimicrobial activity. Due to their lipophilic nature and low molecular weight, these compounds are able to cause structural and functional damage in the cell of microbes by disrupting the membrane permeability and the osmotic balance of the cell (Kalagatur et al. 2015; Prakash et al. 2015; Grata 2016). Moreover, some EOs may inhibit the action of some enzymes, including mitochondrial enzymes such as lactate, malate, and succinate dehydrogenases. These enzymes are involved in ATP biosynthesis (Chen et al. 2013; Nazzaro et al. 2017) as well as H^+^-ATPase activity; inhibiting these processes leads to intracellular acidification and cell death (Ahmad et al. 2013). The inhibition of mycelial growth also depends on the concentration of EOs (Dambolena et al. 2010; Gömöri et al. 2013; Sumalan et al. 2013; Kumar et al. 2014; Elgorban et al. 2015; Perczak et al. 2016). In addition, a majority of plant EOs display high biological safety and are classified as “generally recognized as safe” (GRAS) by the Food and Drug Administration (FDA) (Kedia et al. 2014).

Many studies in recent years have focused on the antifungal potential of EOs against *Aspergillus* and *Fusarium* genera, mainly on cereal matrices such as rice, oat, maize or wheat (Fandohan et al. 2004; Marin et al. 2004; Velluti et al. 2004; Sumalan et al. 2013; Esper et al. 2014; Prakash et al. 2015; Kumar et al. 2016; Santamarina et al. 2016; Tagne et al. 2016; Tian et al. 2016; Boukaew et al. 2017; Bozik et al. 2017). However, the available literature provides limited data concerning comprehensive studies on the effect of EOs on the inhibition of both *Fusarium* growth and mycotoxin biosynthesis.

Thus, the main aim of the present study was to determine the antifungal activity of selected EOs and, consequently, mycotoxin biosynthesis inhibition in wheat grain. In our research, we tested (1) the antifungal effect on chosen *Fusarium* species of eight selected EOs of different origin, and (2) the influence of EOs on the growth of two mycotoxigenic *Fusarium* strains (*F. culmorum* and *F. graminearum*) on sterile wheat grain, determined by analysis of the concentrations of ergosterol (as selective fungal indicator), zearalenone, and deoxynivalenol with derivatives (as toxic secondary metabolites of *Fusarium* genus).

## Materials and methods

### Plant material

Wheat grain (Fortuna variety) was obtained from the Department of Pesticide Investigation, Institute of Plant Protection—National Research Institute in Poznań, Poland. The samples of 25 g were mixed with a small amount of water (10 cm^3^ to prevent sample burning during sterilization) in Erlenmeyer flasks and sterilized at 121 °C.

### *Fusarium* strains

*Fusarium graminearum* strain KZF1 (elsewhere referred to as *F. graminearum*) and *F. culmorum* strain KZF5 (elsewhere referred to as *F. culmorum*) were obtained from the collection of the Department of Pesticide Investigation, Institute of Plant Protection—National Research Institute in Poznań, Poland. Tested strains were incubated at 25 °C in Petri dishes (9 cm diameter) on PDA (Potato Dextrose Agar, BioShop, Canada) for 5–7 days.

### Standards, chemicals, and reagents

Group B trichothecenes (deoxynivalenol, 3- and 15-acetyldeoxynivalenol, nivalenol, and fusarenon X), zearalenone, and ergosterol-certified standards and solvents for analysis (HPLC grade) were purchased from Sigma-Aldrich (Steinheim, Germany). All chemicals used for mycotoxin extraction and purification were purchased from POCh (Gliwice, Poland). Water for the HPLC mobile phase and trichothecene extraction was purified using the Milli-Q system (Millipore, Bedford, MA, USA). Trimethylsilyl imidazole, trimethylchlorosilane, and Tween 80 were also purchased from Sigma-Aldrich (Steinheim, Germany).

### Essential oils (EOs)

The objects of the study were eight selected EOs: cinnamon bark (*Cinnamomum zeylanicum*, Indonesia), oregano herb (*Origanum vulgare*, Mediterranean countries), palmarosa leaves (*Cymbopogon martini*, India), orange peel (*Citrus aurantium dulcis*, Brazil), verbena leaves and flowers (*Thymus hiemalis*, Spain), spearmint leaves (*Mentha viridis*, China), fennel seeds (*Foeniculum vulgare dulce*, Russia/Bulgaria), and rosewood (*Aniba rosaeodora*, India). EOs were obtained from Ecospa Rita Kozak-Chaber Artur Chaber s.c., Poland and from Zrób Sobie Krem-Kosmetyki Naturalne Katarzyna Damętka–Zomerfeld, Poland. Solutions of EOs (20% concentration) were prepared by mixing the certified reference material with water and Tween 80 (10%) as an emulsifying agent.

### Evaluation of antifungal activity of EOs

The antifungal effect of tested EOs was determined by disc diffusion assay (El Ouadi et al. 2017; Munhuweyi et al. 2017). First, conidia suspensions were prepared by harvesting the conidia from fresh cultures on Petri plates and mixing with 10 cm^3^ of sterile saline. The conidia concentration was adjusted to approx. 10^6^ conidia/cm^3^ by enumerating using a haemocytometer chamber. The 100 μl conidia suspensions were spread on PDA Petri plates with a sterile glass spreader. Next, 10 μl aliquots of EOs was individually loaded into 6-mm-diameter sterile paper discs (WhatmanTM, USA) and placed on a Petri dish. The fungal cultures with discs soaked with EOs were incubated at 25 ± 2 °C for 5–7 days, depending on the indicator microorganism. After incubation, the inhibition zones were measured. All experiments were performed in triplicate, and the results are presented as an average of three replications.

### Determination of minimum inhibitory concentration

The minimum inhibitory concentration (MIC) of EOs was determined by the microdilution method (Stupar et al. 2014; Gwiazdowski et al. 2018). Serial, twofold dilutions of the EOs (v/v) were prepared in 96-well microtiter plates in PDB (potato dextrose broth). Next, conidia suspensions of *Fusarium* strains, prepared as described above, were introduced into the wells in equal amounts. The plates were incubated at 25 ± 2 °C for 5–7 days, depending on the indicator organism. The MIC was defined as the lowest concentration of EO that completely inhibited visible growth after incubation.

### Effect of EOs on the growth of *Fusarium* isolates and mycotoxin production

The effect of EOs on the growth of *Fusarium* fungi on wheat was investigated using the method described by Shi et al. (2014) with some modifications. Each EO solution (5 cm^3^) was mixed with 25 g of sterile wheat grain in an Erlenmeyer flask. The mixture was vigorously stirred. Then, three rings (6 mm) of solid culture of the pathogen (*F. graminearum* or *F. culmorum*) were added to each Erlenmeyer flask and mixed. Solutions of Tween 80 and deionized water were added to the control samples without the addition of EO. Next, the prepared mixtures were stored in the dark at 25 °C for a period of 28 days. After incubation, samples were dried, milled, homogenized and prepared for chromatographic analysis.

### Chemical analysis

#### Ergosterol (ERG)

Homogenized grain samples (100 mg) were suspended in 2 cm^3^ of methanol in a culture tube and treated with 0.5 cm^3^ of 2 M aqueous sodium hydroxide (Perczak et al. 2016). Tightly sealed samples were irradiated three times in a microwave oven (370 W) for 10 s and then neutralized with 1 cm^3^ of 1 M aqueous hydrochloric acid. Samples were extracted with *n*-pentane (3 × 4 cm^3^) and transferred to the vials. Extracts were evaporated to dryness in a stream of nitrogen. Before analysis, dry residues were dissolved in 1 cm^3^ of methanol. Twenty microlitres of the prepared mixture was analyzed by HPLC. The ERG separation was performed on a 3.9 mm Nova Pak C-18, 4 mm column with methanol:acetonitrile (90:10, v/v) as the mobile phase at a flow rate of 1.0 cm^3^/min. ERG was detected with a Waters 2996 Photodiode Array Detector (Waters Division of Millipore, Milford, MA, USA) set at 282 nm. The presence of ERG was confirmed by a comparison of retention times with the external standard and by co-injection of every tenth sample with an ERG standard. The detection limit was 10 ng/g.

#### Zearalenone (ZEA)

Plant material (5 g) was homogenized for 3 min with 5 cm^3^ of acetonitrile:water (90:10, v/v) solution. ZEA was extracted and purified on a Zearala Test column (Vicam, Milford, CT, USA) according to a previously described procedure (Goliński et al. 2010). The elute was evaporated to dryness at 40 °C under a stream of nitrogen. The dry residue was stored at − 20 °C until HPLC analysis. Extracts were dissolved in a 500 cm^3^ mixture of acetonitrile:methanol:water (70:20:10, v/v/v), homogenized in an ultrasonic bath (Ultron, type U-505, Dywity, Poland), filtered through a syringe filter of 0.2 µm mesh size and transferred to the chromatographic column. The chromatographic system used in the study consisted of a Waters 2695 high-performance liquid chromatograph (Waters, Milford, CT, USA) with Waters 2475 Multi *λ* Fluorescence Detector (*λ*_ex_ = 274 nm, *λ*_em_ = 440 nm) and Waters 2996 Photodiode Array Detector and a Nova Pak C-18 column (150 × 3.9 mm). Data were processed using Empower 1 software (Waters, Milford, CT, USA). Quantification of ZEA was performed by measuring the peak areas at the retention time according to the relevant calibration curve. A Photodiode Array Detector (PDA) was used to confirm the presence of ZEA based on the characteristic spectra of this compound. The limit of detection was 1.0 ng/g.

#### Trichothecenes

Group B trichothecenes were extracted from plant material according to Perkowski et al. (2003). Type B trichothecenes (deoxynivalenol (DON), 3-acetyldeoxynivalenol (3-AcDON), 15-acetyldeoxynivalenol (15-AcDON), nivalenol (NIV), and fusarenon X (FUS-X)) were analyzed as trimethylsilyl derivatives using an external standard. Trimethylsilyl derivatives were obtained through the reaction with 100 μl of trimethylsilyl imidazole and trimethylchlorosilane (100:1, v/v) mixture and run in a 10 cm^3^ vial at room temperature for 20 min. Chromatographic separation and the analysis of group B trichothecenes were carried out using a gas chromatograph (Varian 450-GC) coupled with a mass detector (Varian 320-MS). The apparatus was equipped with an autosampler (CP-8400) and a capillary column (Varian SLB-5MS, 0.25 mm × 30 m). Samples of 1 μl were injected into the injector chamber at 280 °C without stream division at the separator temperature of 290 °C. The total time of analysis was 24.47 min. Multiple reaction monitoring (MRM) was carried out for trichothecenes, and the retention times for the above toxins (DON, FUS-X, 3-AcDON, 15-AcDON, and NIV) were 13.16, 14.35, 14.42, 14.59, and 14.72 min, respectively. The flow rate for helium was 0.7 cm^3^/min. The results were subjected to processing in the Varian MS Workstation ver. 6.9.2 software. The limit of detection for each analyzed mycotoxin (DON, 3- and 15-AcDON, NIV, and FUS-X) was 1 ng/g.

### Statistical analysis

The presented results are the means (± standard deviation) of three replicate trials. The effect of EOs on the reduction of ergosterol, zearalenone, and group B trichothecenes was examined by multivariate analysis of variance (ANOVA). Analyses were carried out using STATISTICA for Windows version 10.

## Results

### Inhibitory effect of EOs on *Fusarium* growth

**Fig. 1:**
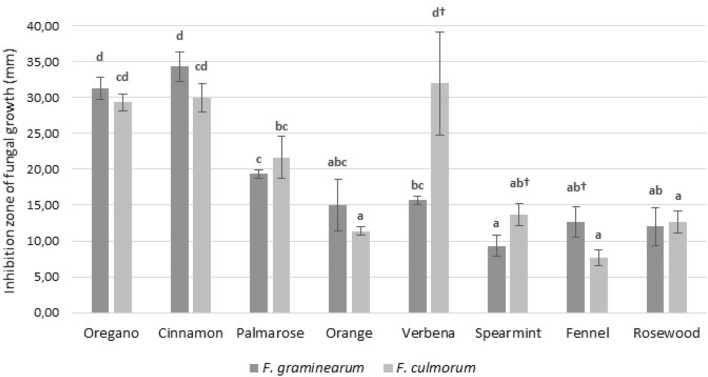
The effect of essential oils on the growth of *Fusarium* strains. *Average with different letters (a–d) for each fungi are significantly different at the *p* < 0.05. **Average with different symbols (†) for each essential oils are significantly different at the *p* < 0.05

Conducted laboratory trials demonstrated the antifungal properties of tested EOs. The results concerning growth inhibition of *Fusarium* isolates tested by diffusion assay are presented in Fig. 1. The highest antifungal activity was demonstrated by cinnamon, oregano, and palmarosa EOs. Spearmint, fennel, rosewood, and orange EOs showed similar and much lower efficacy in the inhibition of tested fungi. It is worth noting that the indicator microorganisms chosen for the study demonstrated different susceptibilities to different EOs. *F. culmorum* exhibited higher sensitivity to the oregano, cinnamon and verbena EOs, while *F. graminearum* was more susceptible to the oregano and cinnamon EOs. Differences in the sensitivity of the tested fungi to EOs were observed only for fennel, spearmint and verbena EOs.

The antifungal effect of each tested EO on the phytopathogenic fungi isolates was expressed in MIC values (Table 1). The oregano, cinnamon, palmarosa, and verbena EOs showed the highest activity, which was in line with the results obtained by the diffusion method. However, considering the sensitivity of the tested fungi, differences between the methods were observed. *F. graminearum* had higher sensitivity to a higher number of EOs (spearmint, rose wood, orange, and palmarosa), while the other oils showed the same activity against both tested fungi. However, it could be noted that the differences in MIC values were sometimes very small; therefore, they were probably not observed in the diffusion method (for example, the MIC difference between rosewood and palmarosa EOs).

### Effect of EOs on the growth and mycotoxin production of the *Fusarium* species

**Fig. 2:**
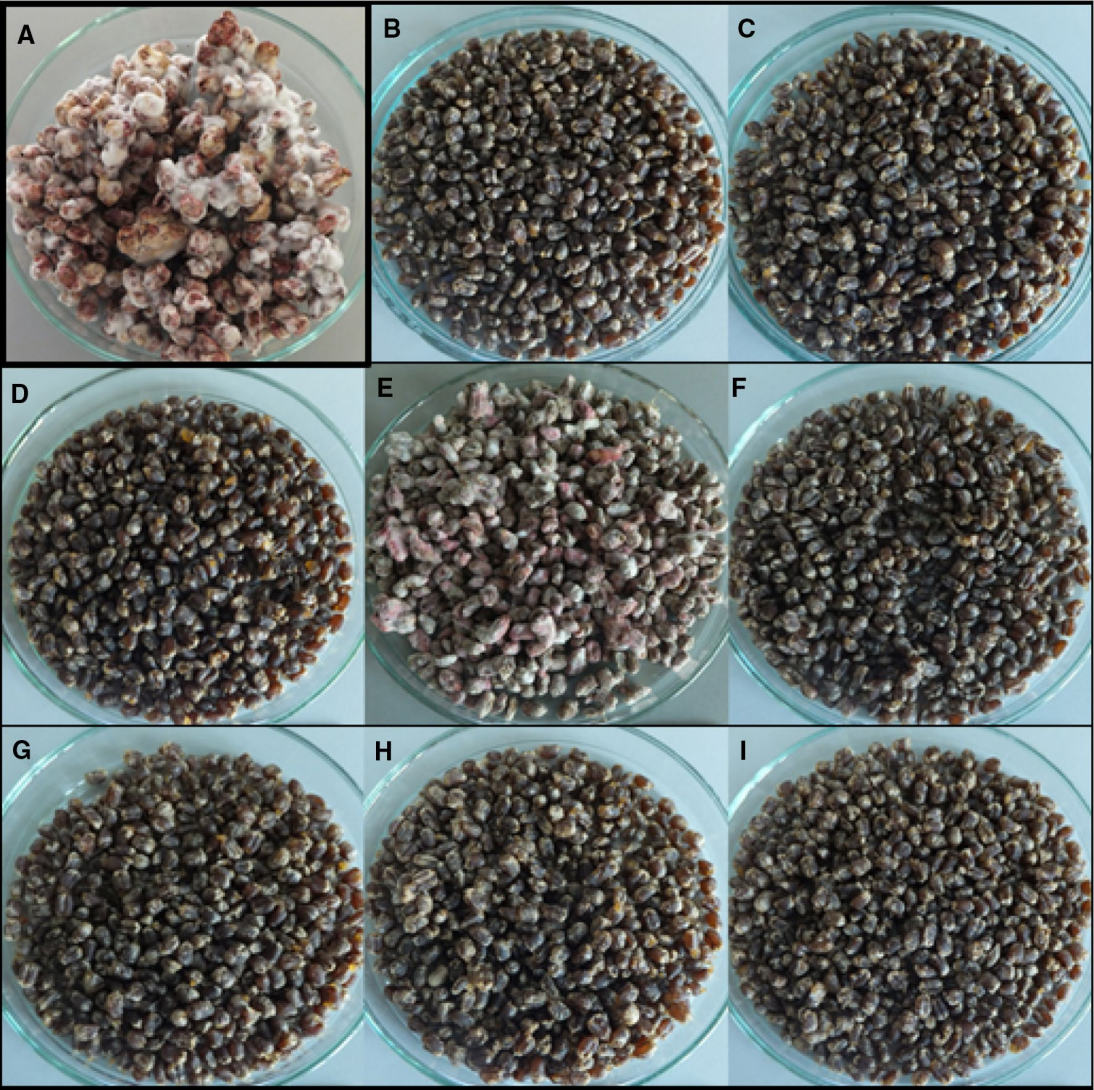
Effect of the application of EOs on the growth of *F. graminearum* on the wheat grain (**a**—control, **b**—oregano oil, **c**—cinnamon oil, **d**—palmarosa oil, **e**—orange oil, **f**—verbena oil, **g**—spearmint oil, **h**—fennel oil, **i**—rosewood oil)

**Fig. 3:**
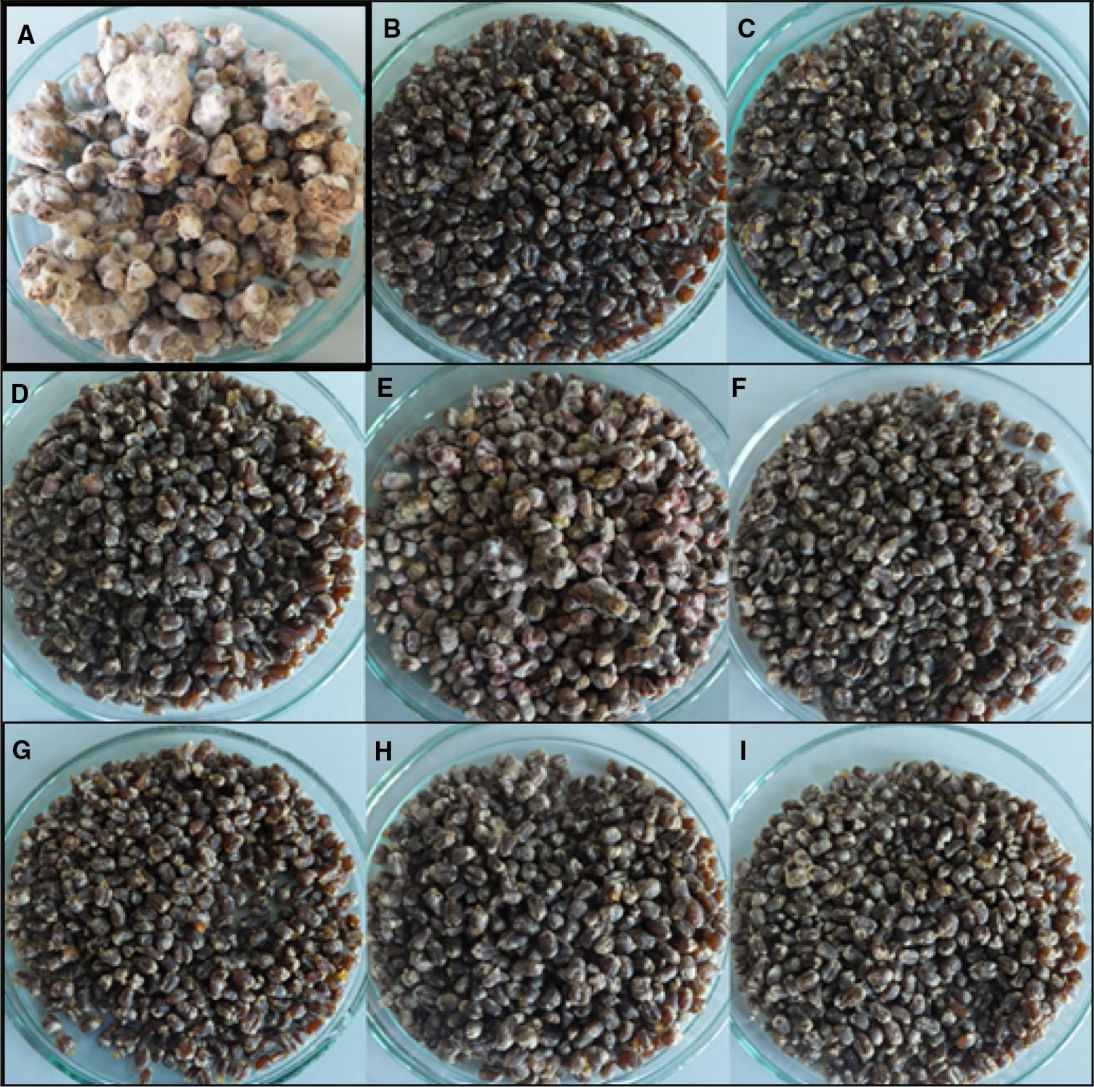
Effect of the application of EOs on the growth of *F. culmorum* on the wheat grain (**a**—control, **b**—oregano oil, **c**—cinnamon oil, **d**—palmarosa oil, **e**—orange oil, **f**—verbena oil, **g**—spearmint oil, **h**—fennel oil, **i**—rosewood oil)

After incubation of the grain samples with added EOs inoculated with *Fusarium* cultures, changes in the growth of *F. graminearum* and *F. culmorum* mycelium in relation to control trials were observed (Figs. 2, 3). The differences in the growth of tested fungi were visible. All EOs reduced the growth of mycelium; however, the weakest effect on the growth inhibition was demonstrated by orange oil. Further, the concentration of ergosterol and *Fusarium* mycotoxins was determined by HPLC analysis to confirm the effect of EOs on fungal growth (Tables 2, 3, 4, 5).

**Table 1:** Minimal inhibitory concentration (MIC) value of essential oils

Essential oils	Minimal inhibitory concentration of EOs (µl/cm^3^)	
	*F. graminearum*	*F. culmorum*
Oregano	< 0.8	< 0.8
Cinnamon	< 0.8	< 0.8
Palmarosa	0.8	3.1
Orange	12.5	> 100
Spearmint	12.5	50
Verbena	< 0.8	< 0.8
Fennel	< 0.8	> 100
Rosewood	6.2	12.5

#### The effect of EOs on the ergosterol concentration in wheat samples

Analysis of ergosterol (ERG) concentration allowed us to estimate the degree of growth inhibition of the two species, *F. graminearum* and *F. culmorum*, in wheat grain after the addition of the EO solutions. The percentage reduction was also calculated in comparison with the control trials (Table 2). The results showed that the concentration of ergosterol was significantly decreased in samples with the addition of tested EOs. An exception was observed in orange EO trials, where the reduction in ERG concentration amounted to 90.99% and 68.13% in *F. graminearum* and *F. culmorum* samples, respectively.

**Table 2:** Ergosterol (ERG) content [µg/g] and percentage of reduction [%] in wheat samples treated with EOs (20% concentration) after inoculation with *Fusarium* species

EOs	ERG content [µg/g] and percentage of reduction [%]			
	*F. graminearum*		*F. culmorum*	
	µg/g	%	µg/g	%
Control (without EOs)	2805.87^a^ ± 744.49	–	3572.26^a^ ± 493.60	–
Oregano	0.83^b^ ± 0.05	99.97	0.54^b^ ± 0.06	99.98
Cinnamon	2.95^b^ ± 0.60	99.89	6.80^b^ ± 8.49	99.81
Palmarosa	0.76^b^ ± 0.25	99.97	0.87^b^ ± 0.06	99.98
Orange	252.80^b^ ± 86.05	90.99	1138.57^b^ ± 1359.37	68.13
Spearmint	0.51^b^ ± 0.09	99.98	0.62^b^ ± 0.28	99.98
Verbena	0.60^b^ ± 0.30	99.98	0.63^b^ ± 0.32	99.98
Fennel	0.39^b^ ± 0.02	99.99	0.71^b^ ± 0.13	99.98
Rosewood	0.68^b^ ± 0.10	99.98	0.45^b^ ± 0.07	99.99

Data were analyzed by Tukey’s test at *p* < 0.05 (a, b—significantly different)

#### The effect of EOs on the zearalenone concentration in wheat samples

The amount of zearalenone (ZEA) in wheat samples inoculated with *Fusarium* isolates was significantly reduced by EO activity/addition (Table 3). Very low concentrations of ZEA (0.00–5.33 µg/g) were observed in *F. graminearum* samples treated with EOs. ZEA reduction was at 99.57–100%, with the lowest efficiency in samples with orange oil. The addition of EOs to the samples inoculated with *F. culmorum* also resulted in a significant reduction in the ZEA amount. The degree of toxin reduction reached 99.08–99.99% with the exception of the sample with orange oil, where the reduction amount was 68.33%.

**Table 3:** Zearalenone (ZEA) content [µg/g] and percentage of reduction [%] in wheat samples treated with EOs (20% concentration) after inoculation with *Fusarium* species

EOs	ZEA content [µg/g] and percentage of reduction [%]			
	*F. graminearum*		*F. culmorum*	
	µg/g	%	µg/g	%
Control (without EOs)	1244.71^a^ ± 231.84	–	117.51^a^ ± 7.88	–
Oregano	nd*^b^	100.00	1.08^b^ ± 0.44	99.08
Cinnamon	0.01^b^ ± 0.01	100.00	0.06^b^ ± 0.02	99.95
Palmarosa	nd^b^	100.00	0.03^b^ ± 0.01	99.98
Orange	5.33^b^ ± 0.36	99.57	37.21^b^ ± 56.17	68.33
Spearmint	nd^b^	100.00	0.34^b^ ± 0.01	99.71
Verbena	nd^b^	100.00	0.03^b^ ± 0.01	99.97
Fennel	0.01^b^ ± 0.00	100.00	0.02^b^ ± 0.01	99.98
Rosewood	nd^b^	100.00	0.01^b^ ± 0.01	99.99

Data were analyzed by Tukey’s test at *p* < 0.05 (a, b—significantly different)

*nd—not detected

#### The effect on group B trichothecene concentration in wheat samples

GC–MS/MS analysis led to the identification of group B trichothecenes in the analyzed wheat grain samples. In the control group inoculated with *F. graminearum,* DON, FUS-X, 3-AcDON, and 15-AcDON were identified, while NIV was not detected (Table 4). The highest concentration was recorded for DON (13.23 µg/g), while the amount of other identified compounds was very low. The addition of EOs resulted in a significant reduction in identified toxin concentrations. The percentage reduction degree reached from 96.33 to 100% depending on the EO used and the mycotoxin. Similarly, inoculation with *F. culmorum* resulted in the formation of trichothecenes in wheat grain (Table 5). DON, FUS-X, and 3-AcDON were detected, while there were no traces of 15-AcDON and NIV. *F. graminearum* produced the largest amounts of DON (5.47 µg/g) in comparison to other identified mycotoxins. The addition of EOs significantly reduced the concentrations of these compounds, and the degree of reduction varied from 94.51 to 100%.

**Table 4:** Trichothecenes (DON, NIV, 3-AcDON, 15-AcDON, FUS-X) content [µg/g] and percentage of reduction [%] in wheat samples treated with EOs (20% concentration) after inoculation with *Fusarium graminearum*

Trichothecenes content [µg/g] (percentage of reduction—%)					
EOs	DON	NIV	3-AcDON	15-AcDON	FUS-X
Control (without EOs)	13.23^a^ ± 13.93	nd*^a^	1.54^a^ ± 0.07	1.57^a^ ± 0.02	1.26^a^ ± 0.07
Oregano	0.04^a^ ± 0.00 (99.70)	nd^a^ –	0.03^b^ ± 0.02 (98.05)	0.03^b^ ± 0.03 (98.09)	0.04^b^ ± 0.00 (96.82)
Cinnamon	0.04^a^ ± 0.00 (99.70)	nd^a^ –	0.04^b^ ± 0.00 (97.40)	nd^b^ (100.00)	0.04^b^ ± 0.00 (96.82)
Palmarosa	0.05^a^ ± 0.00 (99.62)	nd^a^ –	0.03^b^ ± 0.03 (98.05)	0.05^b^ ± 0.00 (96.82)	0.03^b^ ± 0.02 (97.62)
Orange	0.05^a^ ± 0.00 (99.62)	nd^a^ –	0.03^b^ ± 0.03 (98.05)	0.02^b^ ± 0.03 (98.73)	0.04^b^ ± 0.00 (96.82)
Spearmint	0.05^a^ ± 0.00 (99.62)	nd^a^ –	0.05^b^ ± 0.00 (96.75)	nd^b^ (100.00)	0.04^b^ ± 0.00 (96.82)
Verbena	0.04^a^ ± 0.00 (99.70)	nd^a^ –	0.05^b^ ± 0.00 (96.75)	nd^b^ (100.00)	0.05^b^ ± 0.00 (96.03)
Fennel	0.05^a^ ± 0.00 (99.62)	nd^a^ –	0.05^b^ ± 0.00 (96.75)	nd^b^ (100.00)	0.04^b^ ± 0.00 (96.82)
Rosewood	0.04^a^ ± 0.00 (99.70)	nd^a^ –	0.04^b^ ± 0.00 (97.40)	nd^b^ (100.00)	0.02^b^ ± 0.03 (98.41)

Data were analyzed by Tukey’s test at *p* < 0.05 (a, b—significantly different)

*nd—not detected

**Table 5:** Trichothecenes (DON, NIV, 3-AcDON, 15-AcDON, FUS-X) content [µg/g] and percentage of reduction [%] in wheat samples treated with EOs (20% concentration) after inoculation with *Fusarium culmorum*

Trichothecenes content [µg/g] (percentage of reduction—%)					
EOs	DON	NIV	3-AcDON	15-AcDON	FUS-X
Control (without EOs)	5.47^a^ ± 1.78	nd^a^	1.60^a^ ± 0.19	nd^a^	0.83^a^ ± 0.73
Oregano	0.04^b^ ± 0.00 (99.26)	nd^a^ –	0.04^b^ ± 0.02 (97.50)	nd^a^ –	0.04^a^ ± 0.00 (97.50)
Cinnamon	0.04^b^ ± 0.01 (99.26)	nd^a^ –	0.05^b^ ± 0.01 (96.88)	nd^a^ –	0.04^a^ ± 0.01 (95.18)
Palmarosa	0.05^b^ ± 0.01 (99.26)	nd^a^ –	0.04^b^ ± 0.01 (97.50)	nd^a^ –	0.03^a^ ± 0.02 (96.39)
Orange	0.04^b^ ± 0.02 (99.26)	nd^a^ –	0.04^b^ ± 0.01 (97.50)	nd^a^ –	0.02^a^ ± 0.02 (97.59)
Spearmint	0.04^b^ ± 0.00 (99.26)	nd^a^ –	0.04^b^ ± 0.00 (97.50)	nd^a^ –	nd^a^ (100.00)
Verbena	0.03^b^ ± 0.00 (99.45)	nd^a^ –	nd^b^ (100.00)	nd^a^ –	0.04^a^ ± 0.00 (95.18)
Fennel	0.03^b^ ± 0.00 (99.45)	nd^a^ –	0.04^b^ ± 0.00 (97.50)	nd^a^ –	0.03^a^ ± 0.00 (96.39)
Rosewood	0.04^b^ ± 0.00 (99.26)	nd^a^ –	0.05^b^ ± 0.00 (96.88)	nd^a^ –	0.05^a^ ± 0.01 (93.98)

nd—not detected

Data were analyzed by Tukey’s test at *p* < 0.05 (a, b—significantly different)

## Discussion

In recent decades, there has been an increasing demand to reduce the use of chemical substances in the plant protection and food industries; therefore, natural compounds such as EOs have been intensively studied. The present study focused on the effect of some EOs on the growth and mycotoxin production of *Fusarium* species based on in vitro experiments, including disc diffusion assays, MIC determination and model experiments in wheat grain. All tested EOs inhibited the growth of *Fusarium* strains; however, the degree of inhibition and MIC value depended on the examined EO and fungal strain. These results are in line with the observations of other authors. Seseni et al. (2015) examined the effect of different EOs on growth inhibition of *Fusarium* under in vitro conditions. The antifungal activity depended on the kind of EO and its concentration. Of the ten examined EOs, clove, thyme and lemongrass demonstrated the highest activity, completely inhibiting the growth of the four tested *Fusarium* species (*F. oxysporum* and three strains of *F. circinatum*) at a concentration of 1000 µl/l. The lowest reduction was observed for a combination of mandarin, grapefruit, and orange EOs, which caused a reduction in mycelium growth up to 25%. EOs with the highest activity were also tested to determine MIC for the fungi. MIC values were differentiated depending on the EO and the *Fusarium* species. The lowest MIC value was determined for lemongrass oil, which inhibited the growth of *F. oxysporum* and one strain of *F. circinatum* at concentrations of 300 µl/l and 400 µl/l, respectively. The MIC of clove and thyme oils were 400–500 µl/l for both tested species. Matusinsky et al. (2015) tested five different EOs for their growth inhibition capabilities on *Fusarium culmorum* strains under in vitro conditions. The addition of 1 µl/cm^3^ of *Thymus vulgaris* essential oil (EO) resulted in complete growth inhibition of these strains. After the addition of oils from *Pimpinella anisum*, *Pelargonium odoratissimum,* and *Foeniculum vulgare*, total growth inhibition with a dose of 5 µl/cm^3^ was obtained, while in the case of *Rosmarinus officinalis* EO, even a dose of 10 µl/cm^3^ was not enough to completely inhibit the two strains of *F. culmorum*. Kalagatur et al. (2015) showed that the EO of *Ocimum sanctum* L. inhibited the growth of *F. graminearum* and the minimum inhibitory concentration (MIC) was 1250 µg/cm^3^. Naeini et al. (2010) tested the antifungal activity of EOs of five medicinal plants (*Zataria multiflora*, *Heracleum persicum*, *Pinaceae*, *Cuminum cyminum,* and *Foeniculum vulgare*) against different *Fusarium* isolates and stated that the MIC values were very differentiated. Depending on the fungal strain and tested EO, the MIC ranged from 63 to 4500 µg/cm^3^. The differences between EOs and the sensitivity of particular *Fusarium* species were also observed by Zabka et al. (2009). The authors observed total inhibition of *F. oxysporum* and *F. verticillioides* with EO obtained from *Pimenta dioica* (L.) and inhibition exceeded 98% with EO obtained from *Thymus vulgaris* at a concentration of 1 µl/cm^3^, while other tested EOs demonstrated weaker antifungal activity at this concentration. The MIC values determined for the five EOs with the highest antifungal properties were established at a level of 0.5–6.7 µl/cm^3^, depending on the tested EO and *Fusarium* strain, which were in agreement with the results obtained in the presented work.

The inhibition of *F. graminearum* and *F. culmorum* growth by tested EOs was confirmed by HPLC analysis of ergosterol content in wheat samples infected by *Fusarium* strains. Ergosterol is a characteristic component of the fungal cell wall, while bacterial, plant and animal cells are devoid of this compound (Weete and Gandhi 1996; Weete et al. 2010). Therefore, ergosterol is considered to be a suitable marker for estimating fungal biomass in different matrices, such as plant material (Richardson and Logendra 1997; Gutarowska and Zakowska 2010; Porep et al. 2014), grass seeds (Richardson and Logendra 1997), soil (Montgomery et al. 2000; Ruzicka et al. 2000), and grains (Saxena et al. 2001; Olsson et al. 2002; Pietri et al. 2004). Yamamoto-Ribeiro et al. (2013) showed that EO obtained from *Zingiber officinale* inhibited the growth of *Fusarium verticillioides*. The concentrations of ergosterol and fumonisin B_1_ were reduced. Additionally, components of EOs are known to inhibit fungal growth. Gao et al. (2016) revealed that thymol inhibited the growth of *F. graminearum*, which was documented by the results of ergosterol analysis. In comparison with the control, the addition of thymol resulted in a reduction of ERG by 25, 50, and 55% for doses of 25, 50, and 100 µg/cm^3^, respectively. In the present work, a significant reduction in ergosterol content was observed in all wheat samples treated with EOs; however, orange oil was the least effective. The literature data suggest that antifungal compounds, including EOs, may inhibit the cell growth of fungi by interrupting ergosterol biosynthesis, which affects cell growth and proliferation (Ahmad et al. 2011). Yamamoto-Ribeiro et al. (2013) determined the ergosterol content produced by *F. verticillioides* treated with different concentrations of ginger EO. Higher concentrations (4000–5000 µg/cm^3^) of ginger EO effectively inhibited ergosterol production, while lower concentrations (500–3000 µg/cm^3^) caused oscillations in ergosterol production. The authors also observed an increase in ERG biosynthesis at 1000 µg/cm^3^. Similar oscillations in ergosterol production were observed by Dambolena et al. (2010) and Lucini et al. (2006).

Although ergosterol is a good marker of fungal growth, it cannot be used as a suitable indicator of mycotoxin content. Stanisz et al. (2015) stated that even a low content of ergosterol did not indicate a low level of mycotoxins. This could be because the death of fungi causes a decrease in ergosterol content, but the amount of mycotoxins usually stays at a constant level. Moreover, mycotoxins are not produced by every fungal strain. In the present work, an almost total reduction in zearalenone and group B trichothecenes was observed in the presence of the majority of EOs, so it was difficult to state the correlation between ergosterol content and the concentration of mycotoxins. Only in the case of orange oil was the reduction of ergosterol and zearalenone content lower in comparison to the reductions with other EOs, and the reduction in ZEA content was proportionally lower than that in samples treated with other EOs.

The effect of EOs on mycotoxin biosynthesis has been observed by many authors on both synthetic media and cereal matrices. Yamamoto-Ribeiro et al. (2013) observed significant inhibition of the production of fumonisin B1 by *F. verticillioides* in liquid medium at a concentration of 4000 µg/cm^3^ and complete inhibition at a concentration of 5000 µg/cm^3^. Kalagatur et al. (2015) studied the effect of the EO from *Ocimum sanctum* L. on the growth and ZEA production of *F. graminearum* in corn grain. The concentration of ZEA significantly decreased with increasing EO concentration. Moreover, at concentrations 1500 and 2000 µg/g of EO, the toxin content was below the limit of detection. The results confirm that EOs may strongly decrease the level of mycotoxins, and the decrease depends on the kind of EO and its concentration. Some authors indicate that the effect of EOs on the production of mycotoxins by *Fusarium* spp. or other fungi is affected by the treatment conditions, such as the temperature and moisture content of the grains. Temperature, dose and type of EOs influenced fumonisin production by *F. proliferatum*, as described by Velluti et al. (2004). In the work of Sumalan et al. (2013), decreased content of DON and FB_1_ was observed under constant conditions of water activity and temperature.

It is worth noting that EOs may also degrade mycotoxins, which may also cause a reduction in their concentration in different matrices. Xing et al. (2014) tested some EOs for the reduction of mycotoxin concentration under in vitro conditions. The best properties were exhibited by cinnamon and lemon oils, which resulted in the reduction of FB_1_ by 66.65 and 53.19%, respectively. Moreover, the influence of incubation time and temperature on the reduction of mycotoxin by cinnamon oil was examined. The results showed that the degree of reduction of mycotoxin increased with incubation time, and after reaching 120 h, a 72.92% reduction was achieved. Additionally, with the increase in the incubation temperature, an increase in the degree of reduction of FB_1_ was observed. Perczak et al. (2016) revealed that EOs reduce zearalenone concentration under in vitro conditions and that the amount of reduction depends on the time of incubation, concentration of the toxin and EO, and pH and temperature conditions. The highest reduction level was reached after 72 h in the case of lemon oil (46.46%). Doubling the dose of EOs (from 100 to 200 µl/cm^3^) resulted in significant differences in the reduction of mycotoxin using white grapefruit EO (from 15.15 to 70.81%), while the addition of lemon oil caused a decrease in the reduction (from 66.56 to 26.97%). Increasing the dose of the toxin from 0.5 to 5.0 µg/cm^3^ resulted in the highest degree of reduction for palmarosa oil (97.89–99.29% for three different pH values) and lemon oil (87.93–97.28%). The use of different pH values did not result in significant differences in the values of reduced concentrations of the ZEA at an initial dose of 0.5 µg/cm^3^, but using a dose of 5 µg/cm^3^, there were differences—the addition of eucalyptus oil resulted in an increase in the degree of reduction at the level of 59.56–91.74%, with increasing pH.

## Conclusions

*Fusarium* species may cause severe plant diseases and produce mycotoxins, which have a serious impact on human and animal health. The EOs inhibited the growth of fungi and reduced mycotoxin production. The results suggest the possibility of EO application as an alternative to chemical pesticides, for example, as a seed treatment intended for sowing, which may contribute to increased resistance of plants to *Fusarium*. Consequently, EOs may increase food and feed safety in the food chain. The vast variety of oils offers many possibilities for both application and further research.
